# Breakfast in Human Nutrition: The International Breakfast Research Initiative

**DOI:** 10.3390/nu10050559

**Published:** 2018-05-01

**Authors:** Michael J. Gibney, Susan I. Barr, France Bellisle, Adam Drewnowski, Sisse Fagt, Barbara Livingstone, Gabriel Masset, Gregorio Varela Moreiras, Luis A. Moreno, Jessica Smith, Florent Vieux, Frank Thielecke, Sinead Hopkins

**Affiliations:** 1 Institute of Food and Health, University College Dublin, Dublin D04 V1W8, Ireland; 2 Department of Food, Nutrition & Health, University of British Columbia, Vancouver, BC V6T 1Z4, Canada; susan.barr@ubc.ca; 3 Nutri Psy Consult, 91 rue de la Sante, 75013 Paris, France; bellisle@uren.smbh.univ-paris13.fr; 4 Center for Public Health Nutrition, University of Washington, Seattle, WA 98195-3410, USA; adamdrew@uw.edu; 5 Division for Risk Assessment and Nutrition, The National Food Institute, Technical University of Denmark, 2800 Kgs. Lyngby, Denmark; sisfa@food.dtu.dk; 6 Nutrition Innovation Centre for Food and Health, Ulster University, Coleraine BT52 1SA, UK; mbe.livingstone@ulster.ac.uk; 7 Cereal Partners Worldwide, CH-1350 Orbe, Switzerland; rdmassetga@nestle.com (G.M.); sinead.hopkins@rd.nestle.com (S.H.); 8 Department of Pharmaceutical and Health Sciences, Faculty of Pharmacy, CEU San Pablo University, 28668 Madrid, Spain; gvarela@ceu.es; 9 GENUD (Growth, Exercise, Nutrition and Development) Research Group, Universidad de Zaragoza, Instituto de Investigación Sanitaria Aragón (IIS Aragón), Instituto Agroalimentario de Aragón (IA2), Centro de Investigación Biomédica en red Fisiopatología de la Obesidad y Nutrición (CIBEROBN), 50009 Zaragoza, Spain; lmoreno@unizar.es; 10 Bell Institute of Health, Nutrition and Food Safety, General Mills, Minneapolis, MN 55427-3870, USA; Jessica.Smith@genmills.com; 11 MS-Nutrition, 13385 Marseille cedex 5, France; florent.vieux@ms-nutrition.com; 12 Thielecke Consultancy, CH-4123 Allschwil, Switzerland; frank.b.thielecke@gmail.com

**Keywords:** breakfast, breakfast nutrients, breakfast foods, breakfast guidelines

## Abstract

Breakfast is often referred to as the most important meal of the day and in recent years has been implicated in weight control, cardio-metabolic risk factors and cognitive performance although, at present, the literature remains inconclusive as to the precise health benefits of breakfast. There are extensive reports of breakfast’s contributions to daily food and nutrient intakes, as well as many studies that have compared daily food and nutrient intakes by breakfast consumers and skippers. However, significant variation exists in the definitions of breakfast and breakfast skippers, and in methods used to relate breakfast nutrient intakes to overall diet quality. The present review describes a novel and harmonised approach to the study of the nutritional impact of breakfast through The International Breakfast research Initiative involving national dietary survey data from Canada, Denmark, France, Spain, the UK and the USA. It is anticipated that the analysis of such data along harmonised lines, will allow the project to achieve its primary goal of exploring approaches to defining optimal breakfast food and nutrient intakes. Such data will be of value to public health nutrition policy-makers and food manufacturers and will also allow consistent messaging to help consumers to optimize food choices at breakfast.

## 1. Introduction

At different times throughout history, those who shape policy have had varying beliefs about the importance of breakfast in overall diet quality. Today, there would appear to be universal recognition that breakfast should play a significant role in helping consumers attain an optimal nutritional profile. There is a wealth of data on the patterns of breakfast intake from national dietary surveys, on the foods commonly consumed at breakfast, their contribution to breakfast intake and on the role of breakfast in meeting overall daily nutrient targets. Notwithstanding the scale of this literature, significant challenges exist given the variation in such basic concepts as defining breakfast or breakfast skippers as well as exploring how breakfast food and nutrient intakes might be related to overall dietary quality. The International Breakfast Research Initiative (IBRI) was established to provide a cross country harmonised analysis of national and large regional food consumption databases to provide a clearer picture of food and nutrient intakes at breakfast. The international collaborative project represents a broad range of expertise from Europe and North America, including behavioral, public health and nutrition scientists from academia and industry. Data from the US, Canada, the UK, Denmark, Spain and France will be used, and it is expected that the provision of such data might permit a more learned approach to defining nutritional quality standards for breakfast. This will involve the development of novel concepts of defining dietary guidelines pertaining to a specific meal, breakfast, rather than to the overall daily food pattern and will facilitate the development of nutritional standards for breakfast by national and international agencies. This will also help to provide clear guidelines to food manufacturers for developing healthy breakfast options and will ultimately benefit consumers in making decisions about breakfast food choices.

## 2. Breakfast: A Brief History

From a physiological perspective, breakfast is unique among our meals in that it is eaten after the longest of our postprandial fasts, in this instance an overnight fast. In his book, A History of Breakfast, Andersen describes some of the features of breakfast in the human diet over centuries. [1] From the gastronomic perspective, breakfast was traditionally, dating back to Greek and Roman breakfasts (ariston and Ientaculum respectively), the least demanding of our meals in culinary terms in that it consisted for centuries as a simple meal of bread, cheese, honey, oil and maybe wine. Thus, it was from the very early times, a meal characterised by convenience since none of these ingredients required preparation or further cooking. Convenience was important for early risers who had to attend crops and livestock and it was also important for those who had to travel distances after rising. In general, breakfast was seen in the earliest days of recorded history and up to the middle ages as a meal for infants and the infirm, but it was acceptable for the labouring classes. By the 16th century, Europeans began to see breakfast as an important meal in terms of overall health. Thomas Cogan, an English school master in Manchester, in describing the skipping of breakfast, argued that to “suffer hunger long filleth the stomach with ill humours”. Whilst breakfast meant simple foods for the masses, including gruels based on oats, rice and other cereals, the rich and ennobled began to include eggs and meat as a component of breakfast. “This “cooked breakfast”, which was widely adopted in the US, was targeted by the Clean-Living Movement. This Movement primarily advocated against alcohol, tobacco and birth control, but also made dietary recommendations: avoiding meat and coffee, using filtered water, and increasing intake of whole grain foods [2]. Against this background, the Seventh Day Adventists John Harvey Kellogg developed the flaked breakfast cereal in 1894 and in 1945, the Kellogg Company first introduced the cereal box that we know today. At any point in history, the timing and nature of breakfast has varied widely both geographically and socially and to this day, that variability prevails. Of considerable interest in present context is the development of shift work and attendant night work. For the majority of countries for which data are available, about 20% of employees are engaged in night [3].

## 3. Existing Recommendations on Breakfast Consumption

It is frequently stated that breakfast is a very important meal and that it deserves special attention, especially among children and this is borne out by published opinions of international agencies, national governments and non-governmental organisations. One of the most wide-reaching reports is that of the European branch of the World Health Organisation, who conducted a health behaviour survey of over 200,000 male and female schoolchildren, 11–13 and 15 years of age in 39 European states in 2009/2010 [4]. Overall, 61% of 13 year olds consumed a breakfast on each school day while the figure fell to 55% among 15 year olds. In general, breakfast consumption was most common among boys and declined with lower socio-economic status. These data show that about half to one third of children don’t have breakfast every day, although the data does not reveal the actual frequency of breakfast intake. The report notes that regular breakfast consumption is associated with higher intakes of micronutrients, a better diet that includes fruit and vegetables and less frequent use of soft drinks. The report concludes that establishing the most effective programmes and policies to promote breakfast intake across countries is a public health challenge.

The American Heart Association issued a scientific opinion in 2017 on “Meal Timing and Frequency: Implications for Cardiovascular Disease Prevention” [5]. As regards breakfast and nutrient balance they cite a cross-sectional survey of the Bogalusa Heart Study (*n* = 504; 58% women; 70% white; mean age, 23 years [range, 19–28 years]) which demonstrated that 74% of breakfast skippers did not meet two thirds of the Recommended Dietary Allowance for vitamins and minerals compared with 41% of those who consumed breakfast. They also cite US data showing that young adults (age, 20–39 years) who reported skipping breakfast had overall daily diets that were less than optimal in terms of nutrient intake as measured by indices of total daily diet quality (Mean Adequacy Ratio, MAR and Healthy Eating Index HEI) [6,7]. Taking into account the direct evidence linking breakfast intake both to overall dietary patterns and the presence of cardio-metabolic risk factors, the statement concludes: “On the basis of the combined epidemiological and clinical intervention data, daily breakfast consumption among US adults may decrease the risk of adverse effects related to glucose and insulin metabolism. In addition, comprehensive dietary counselling that supports daily breakfast consumption may be helpful in promoting healthy dietary habits throughout the day”.

Most institutes of nutrition and dietetics recommend eating a healthy breakfast as an integral component of a nutritionally optimal diet such as in the US, UK and Australia among many [8,9,10]. Individual governments have issued dietary advice promoting breakfast [8]. Where specific advice exists advocating a healthy breakfast, it is usually focussed on food group choice, which is not normally based on data derived from the analysis of national dietary surveys [11,12,13]. In some countries such as the US and Mexico, detailed policy guidelines exist for both food and nutrient intake for school breakfasts [14,15]. However, in both cases, the nutritional guidelines are (a) adapted from the general dietary guidelines for the population which are then applied to children’s recommended energy intakes; (b) do not relate the food-based advice to any nutritional origins and (c) fail to provide any detailed examination of the role of micronutrients in breakfast other than a bland recommendation that their intake should meet 20% of the RDA. In addition, several breakfast quality indexes have been proposed at national level [16,17,18]. These usually follow very closely diet guidelines by focusing on food groups and some nutrients, without taking into account actual intakes at breakfast.

## 4. Breakfast as a Source of Daily Nutrients

There is a very extensive literature on nutrient intake at breakfast and the extent to which breakfast-based nutrients contribute to overall daily nutrient intake. The vast majority of such studies show a clear overall nutritional benefit of consuming breakfast in respect of the key nutrients of public health importance in many countries. This section seeks to select a few large nationally representative studies in this field to illustrate the impact of breakfast on overall daily nutrient intake.

Australia (2017): Data were obtained from the 2011–2012 National Nutrition and Physical Activity Survey which used a 24-h recall method in a sample of 12,153 subjects. Breakfast patterns were studied in 2821 children and adolescents aged 2 to 18 years of age [19]. Some 9% of the sample were classified as breakfast skippers while the remainder were equally divided between those consuming cereal-based breakfasts (45%) or other breakfast types (46%). Among breakfast skippers, 61% were in the 14–18 year old age group. Breakfast skippers had higher intakes of saturated fatty acids and lower intakes of dietary fibre and most micronutrients. Among breakfast consumers, those consuming breakfast cereals had higher intakes of total sugars but not of either free and added sugars and also had higher intakes of dietary fibre. They had lower intakes of total fat and sodium. Generally, they had higher intakes of most micronutrients.

Brazil (2017)*:* The data used were from the Brazilian National Dietary Survey of 2008–2009 used two 24-h recalls [20]. The total sample surveyed was 34,003 and the data reported on breakfast intakes were for 7276 children and adolescents aged 10–19 years. Breakfast skippers accounted for 7% of the population while occasional and regular breakfast consumers represented 12% and 81% respectively. The mean daily intakes of total energy, sugar, and calcium were higher among occasional consumers and skippers. Breakfast consumers had higher intake of vitamins B_12_, C, and D. Breakfast consumers had lower total and added sugar intakes and lower calcium intakes compared to occasional consumers and skippers.

Canada (2004): Data from the nationally-representative Canadian Community Health Survey of 2004 were used to study the contribution of breakfast to daily nutrient intake and the prevalence of nutrient inadequacy (% with intakes < EAR) of Canadian children (aged 4–18 year; *n* = 12,281) and adults (aged 19+ year; *n* = 19,913) [21,22]. Data were collected using multiple-pass 24-h recalls. Overall, 10% of children and 11% of adults were breakfast non-consumers, 33% of children and 23% of adults consumed breakfasts containing ready-to-eat cereal (RTEC), and 57% of children and 66% of adults consumed other breakfasts. Breakfast non-consumers had lower energy intakes than breakfast consumers; accordingly, reported nutrient intakes were energy-adjusted. Breakfast consumers, and to a greater extent, consumers of RTEC breakfasts, had higher intakes of fibre, several vitamins and minerals, and among children, lower intakes of fat. Among both adults and children, the prevalence of nutrient inadequacy (intakes < EAR) for vitamins A and D, calcium, iron and magnesium were significantly higher among breakfast non-consumers than RTEC breakfast consumers; among children, this was also the case for vitamin B6, folate and zinc. The prevalence of inadequacy among consumers of other breakfasts generally fell in between the other two groups.

Korea (2009): The sample of this study consisted of 1600 children aged 7–18 years attending elementary, middle, or high schools and who participated in the 2001 National Health and Nutrition Survey [23]. A 24 h-record was used to estimate dietary habits. The sample was divided into 4 groups: breakfast skippers and three levels of breakfast intake based on % contribution of breakfast to estimates of energy requirements (EER): low (<10% EER), moderate (10–25% EER) or sufficient (>25% EER). The percentages consuming daily diet with protein, vitamin A, B1, B2, niacin, vitamin C, calcium, phosphorus, or iron less than Estimated Average Requirement decreased in the breakfast groups as %EER increased. Similarly, a Dietary Variety Score which measures overall daily food variety rose with greater contribution of breakfast to daily energy intake.

USA (2018): Food and nutrient intakes were evaluated in a sample of children aged 2–5 years (3443) and 6–12 years (5167) from the NHANES 2005–2012 survey [24]. Diet quality was assessed for breakfast skippers and consumers. Snacks, sweets, beverages (non-dairy, non-alcoholic) and mixed dishes were higher in contribution to daily energy intake among skippers as compared to consumers. Grains and milk products contributed more to total daily energy intake among breakfast consumers. Breakfast eaters consumed lower overall daily levels of added sugar and higher levels of fibre, folate, iron, vitamin C, vitamin A and calcium. For both age groups, the Healthy Eating Index was higher among breakfast consumers compared to non-consumers. Across all meals (breakfast, lunch, dinner, and snacks) intakes of total and saturated fats were lowest at breakfast while those of folate and vitamin A were highest. Some minor differences existed in this regard across the two age groups.

UK (2017): Data are available from the UK National Diet and Nutrition Survey Rolling Programme on breakfast intakes and nutrient patterns in 1686 children and adolescents (11–18 years) using a 4-day diary [25]. Comparisons were made for those never recording a breakfast intake (17%), those consuming breakfast on 1–3 days of study (52%) and those eating breakfast every day (31%). Breakfast consumption was generally associated with higher energy intakes and in turn, with higher intakes of folic and ascorbic acids, fibre, calcium, iron and iodine intakes and lower intakes of total carbohydrates and sodium. Intakes of non-milk extrinsic sugars were lower among daily breakfast consumers compared to non-consumers if breakfast. In general, these absolute differences were sustained when adjusted for energy intake.

The data, illustrative of a large literature on the subject show the importance of breakfast intake in shaping overall optimal nutrient intake. There tends to be an emphasis on comparing breakfast skippers with breakfast consumers and within breakfast consumers to make limited comparisons of nutrient intake consequent to the nature of breakfasts consumed. Thus, in the studies included in the present international project, attempts are made to provide as harmonised an approach as possible to studying the association of breakfast consumption and breakfast skipping on overall nutrient intake. Thus, there is a common focus on nutrients and a common approach to relate breakfast nutrient intake data to the overall diet quality the Nutrient Rich Foods (NRF) index as a measure of overall dietary quality [26] In addition, diet modelling with linear programming will be used to further examine the potential role of breakfast in a nutritionally adequate diet.

Finally, several important factors often not considered in the literature such as energy under-reporting, nutritional supplement use and the use of overall diet quality indices to ascertain the true impact of breakfast on overall nutrient intake are considered in more detail in a later section of this review.

## 5. Food Intake Patterns among Breakfast Consumers

The many studies on the contribution of breakfast to mean daily nutrient intakes also provide data on the main foods consumed at breakfast and their contribution to nutrient intakes both at the level of breakfast and the overall daily nutrient intake. Comparisons across studies are difficult for many reasons. The exact definition of given food categories varies very considerably across studies. A category “regular breads and rolls” maybe used in one study, whereas others might use terms such as “whole grain bread”, “white bread” or “other breads”. Moreover, food intakes may be given in quantitative terms such as intakes in grams at breakfast or for the total day. Similarly, food contributions to breakfast or total daily energy intake may be expressed as a % of total or breakfast energy intake. The following again are examples of the type of food intake data that can be derived using different forms of data analysis and different end points of the impact of food intake patterns at breakfast.

France (2016): A study of 529 children aged 9–11 in the city of Rennes was used with cluster analysis to identify breakfast patterns in French children [27]. Four breakfast patterns were identified: Sweets breakfast (40%), traditional French breakfast (27%), ready-to-eat cereal with milk (18%) and dairy and juice breakfast (10%). The overall quality of breakfasts was determined using the mean adequacy ratio (MAR). The cereal-based breakfast had the highest overall MAR score at 30.2% which fell to 25% for the sweet breakfast, 23% for the traditional French breakfast and to 18% for the dairy and juice breakfast.

Germany (2017): From a sample of 1447 subjects randomly chosen from the EPIC Potsdam cohort, 815 agreed to participate in providing 2, 24-h dietary recalls of which 664 completed the study [28]. A Breakfast Quality Index (BQI) and a Daily Intake Quality Score (DQS) were calculated based on a healthy Eating Index categorisation of foods. Using data on blood lipids, HbA1c and C-reactive protein, a biomarker score (B Score) was calculated. The BQI value was more highly correlated with B Score indicating that the more nutritionally balanced the breakfast was, the more likely an individual was to have a favourable cardio-metabolic profile. The dietary data were subject to principal component analysis (PCA) to establish general patterns of food choice for both breakfasts and the total day. Only one of the breakfast patterns, that of ‘dairy and cereal’ pattern, was positively related to a favourable B Score. As regards daily patterns, only that PCA group classified as the ‘prudent diet pattern’ was indicative of a favourable cardio-metabolic profile.

Mexico (2017): Cluster analysis of the Mexican National Food Consumption Survey with data from 3760 children. These data revealed 6 dietary patterns: breakfast skippers (17%), sweetened beverages (10%), sandwiches and quesadillas (9%), eggs (8%), tortillas and beans (12%), cereal and milk (6%) and milk and sweetened breads (38%) [29]. There was little evidence of any major role for socio-economic factors in determining breakfast choices. Each breakfast choice had its own strength or weakness. For example, the highest added sugars came from the ‘sweetened beverage’ group while the highest intake of fibre came from the ‘tortillas & beans’ group. However, when total daily nutrient intakes were considered, the ‘sweetened beverage’ group’s added sugar intake did not differ significantly from all other breakfast patterns, including those who skipped breakfast. In contrast, the higher fibre intakes at breakfast were reflected in an overall higher daily fibre intake. None of the breakfast groups complied with the Mexican Breakfast School Guidelines.

United States: The 1994–1996 Continuing Survey of Food Intake by individuals involved about 23,700 individuals of which 15,641 adults aged 18–65 were selected for the breakfast analysis [30]. The methodology uses was a 24-h recall. Eggs, cereal, toast, fruit or fruit juices, coffee, and soft drinks were identified as ‘lead’ foods around which breakfast choices were made. Breakfast skipping was also recorded. The categories identified were: (1) eggs plus any other primary food group (13.3% of total breakfasts); (2) cereal plus groups not including eggs (16.8%); bread alone (19.2%); cooked cereal (3.0%); fruit (5.1%); coffee and high fat dessert (15.1%); miscellaneous (4.5%); no breakfast (23.0%). The cereal group and the fruit group had the best nutritional profile in terms of total and saturated fats, fibre and carbohydrate. Cereals however, scored highest for calcium, folate and iron intakes. Socio-economic differences in patterns of breakfast intake were notable. For example, as either income or educational status rose, egg-based breakfasts declined, and cereal based breakfasts rose.

A clear understanding of the contribution of different foods to overall nutrient intake will be essential in attempting to develop locally based guidelines on food choice for an optimal breakfast. That analysis can be confined to the mean derived from all meal choices for breakfasts. In some cases, it can be studied in greater depth if breakfast food choice is broken down into patterns of food choice for breakfast meals through principal component or cluster analysis. Another alternative might be an overall nutritional score for each subject allowing tertiles or quartiles of such an index to be related back to any aspect of breakfast nutrition: nutrient intake, food choices or meal patterns.

## 6. Breakfast and Health Outcomes

At present, a significant proportion of the literature on the benefits of breakfast is focused on health outcomes rather than dietary outcomes, although the two are frequently linked. The main thrust of the present consortium is to develop a greater understanding of the food and nutrient intakes at breakfast and their contribution to overall diet quality across many geographic regions rather than any impact of breakfast on health outcomes. Nonetheless, it is valuable to record some of the main directions that research in this area is making.

Several large prospective studies support the association between breakfast consumption and lower risk of obesity and weight gain [31,32]. Some intervention studies have challenged the importance of breakfast skipping in weight management and other scientific commentaries have cast a more critical view of the overall research approach to this area [33,34]. Support for the association between skipping breakfast and impaired glucose metabolism comes from four prospective studies that link skipping breakfast with a greater risk of type-2 diabetes: The Health Professionals Follow-Up Study, The Nurses’ Health Study, a Japanese study and the German EPIC cohort [28,35,36,37] provide evidence that regular healthy breakfast consumption is associated with improved glycemic control. Several cross-sectional studies reported an inverse association between breakfast consumption and risk factors of cardiovascular disease [28]. A prospective study of US Health Professionals documented an increase in incidence of CVD amongst those North American men who regularly failed to eat breakfast [38]. Similarly, a study, conducted in Japan showed that infrequent breakfast intake was associated with an increased risk for total CVD, total stroke, and for cerebral haemorrhage [39]. However, it was not associated with greater risk of subarachnoid haemorrhage, cerebral infarction, or coronary heart disease in this population. Data from a Spanish cohort PESA (Progression of Early Subclinical Atherosclerosis) further supports the suggestion that skipping breakfast is associated with increased odds of prevalent non-coronary and generalized atherosclerosis independently of the presence of conventional cardiovascular risk factors [40]. Taken together, data from prospective studies support consistent and strong cross-sectional evidence suggesting that breakfast consumption is associated with a reduction of cardio metabolic risk factors.

Breakfast intake is frequently related to mental alertness. Much of the research in breakfast and its potential effects on improving cognitive performance has been conducted in children and adolescents. A recent meta-analysis examined the literature on breakfast consumption versus breakfast skipping and also the nutritional composition of consumed breakfasts [41]. The studies included (*n* = 45) were a mix of acute and chronic breakfast intake. Whilst there was evidence to support improved cognitive function, particularly short-term benefits on attention, executive function, and memory (more pronounced in undernourished children), the authors concluded that there were insufficient studies to draw any firm conclusions. Similarly, a review in adults that included 38 studies reported small effects on memory, but emphasized that strong conclusions are hampered by methodological disparity.

## 7. Challenges in Studying Breakfast Consumption

Notwithstanding the proposed importance of a good breakfast in determining overall nutritional quality, there are major gaps and assumptions in our knowledge base on breakfasts and agreement on certain definitions and research approaches may help to resolve these difficulties. This section outlines the main areas where variations in approaches and understandings may impinge upon how data are gathered, presented and, above all, interpreted.

In the course of this collaborative study, it has become clear that whilst an agreed definition of breakfast is attractive, the reality is that dietary survey methodologies vary quite considerably in how data is collected, such that a universal definition of breakfast becomes impossible. One major US review of the role of breakfast in the overall determination of diet quality, proposed a definition built around the foods consumed as the first meal of the day. The specific definition proposed was: “*Breakfast is the first meal of the day that breaks the fast after the longest period of sleep and is consumed within 2 to 3 h of waking; it is comprised of food or beverage from at least one food group and may be consumed at any location*” [42]. This consensus was reached based on an extensive review of breakfast definitions used in the literature and drew on 8 definitions as proposed in 14 published studies. However, some very large and highly regarded national studies do not offer the subjects any option to define their various eating occasions. Therefore, such surveys must be extensively examined to determine the most suitable definition of breakfast. The earlier the hour at which a breakfast meal might be considered, the more likely it was to pick up late night consumers including quite likely, shift-workers. Equally, the later the cut-off for defining the hour the breakfast intake might cease, the more likely it is to include foods more typical of lunch than breakfast. Thus, an iterative process was needed to identify the most likely period over which breakfast would be consumed. It may well be that a general recommendation to those involved in designing dietary surveys would be to either ask that the respondents choose a term to define the meal consumed, one of which would be breakfast or, at a minimum, to define the hour of rising.

There is considerable interest in the role of consuming and skipping breakfast on health outcomes and also on nutrient related outcomes. The study of breakfast skipping varies according to how such a practice is defined. In studies involving a single 24-h recall, breakfast intake can be readily dichotomized into ‘skippers’ and ‘consumers’. However, in studies of longer duration or of multiple 24-h recalls, subjects may be breakfast skippers if they skip breakfast on all days of the study. Breakfast consumers might be defined by the intake of a breakfast on all survey days. “Irregular” breakfast consumers lie in between and may be breakfast consumers on just one or on several days of the study. There is no agreement as to how to define skippers, irregular and regular breakfast consumers and this contributes to variation in conclusions reached as to the impact of breakfast on overall nutrient intake. This extends to the manner in which total daily nutrient intake is influenced by breakfast intake.

Energy under-reporting is a major phenomenon of all dietary surveys [43]. Objective techniques exist which give a very accurate measure of energy expenditure which in the short term and in a weight stable condition, can be equated to energy intake. Such techniques include at one end the use of stable isotopes such as doubly labelled water or at the other end, heart rate output. A crude estimate of energy requirements can be computed from equations relating age, gender, weight and height to basal metabolic rate which combined with an agreed multiple can yield a crude cut off point to indicate energy under-reporting. In some studies, energy under-reporters are eliminated before the data analysis. In others, energy under-reporting is ignored. Either way, it remains impossible to accurately state on an individual level, which food were under-reported in the estimation of overall energy intake. Under-reporting of a given food may involve under-reporting of the frequency of eating occasions or under-reporting of serving size at a given eating occasion. If skipping breakfast or the selection of some particular type of breakfast can influence energy intake, then it would make sense that comparisons adjusted for energy intake. The exclusion of energy under-reporters from large nationally representative surveys will in effect create the problem of the distortion of demographic balance since under-reporters are at a higher probability of being female, overweight and of lower socio-economic status.

Nutritional supplements are frequently consumed at that time of the day which coincides with breakfast intake and unless accounted for can lead to a significant distortion in the interpretation of nutrient intake patterns. One US study examined the impact of dietary supplements among breakfast consumers and showed that supplement usage significantly improved overall daily intake for vitamins A, B6, C and D and niacin and also improved daily intakes of magnesium, iron and zinc [44]. Breakfast studies should confine their analysis to nutrients derived from breakfast foods and not nutritional supplements. That is not to diminish the importance of the latter in the diets of many people. However, to develop dietary and food guidelines for breakfast, the focus should be on foods.

Given the need to relate the quality of breakfast eating patterns to overall daily nutrient intake, some measure of the overall quality of the daily diet must be used. Knowing that breakfast consumption leads to an overall improvement of mean daily nutrient intakes cannot address the question as to whether it has improved some more global definition of dietary quality. Many such measure of diet quality exist. German studies have developed a breakfast quality index (BQI) which simply measures how food and nutrient intakes at breakfast meet with some predefined standard of intake of selected nutrients [18]. This is useful, but it does not allow breakfast eating or breakfast skipping habits to be related to a global diet quality index. Examples of such include: The Healthy Eating Index, Alternate Healthy Eating Index, MedDietScore, PREDIMED Mediterranean Diet Score, and the Dutch Healthy Diet-Index [45]. Other approaches involve the Mean Adequacy Ratio (MAR) and the Nutrient Rich Food (NRF) index [26] In the present international collaborative study, overall dietary quality will be assessed using the NRF index, drawing on local nutritional standards. The advantage of the NRF is that it relies on the intake of nutrients, instead of food groups such as used in the Healthy Eating Index. Whilst food group data might be valuable for within-database analysis, the very significant variation in the classification of food groups makes this extremely problematic to compare between datasets and countries, and thereby to reach common conclusions.

## 8. Conclusions

The IBRI project is the first trans-Atlantic international collaborative study on the nutritional quality of breakfast that is based representative samples of the population in six different countries. In each country, analyses will establish the frequency of breakfast intake as well as energy and nutrient intakes from breakfast alone and from the total diet. The project objective is to formulate a set of breakfast-specific dietary guidelines that are both nutrient and food based. While similarities in energy intakes at breakfast may exist across countries, there is also considerable diversity in the local choices of breakfast foods and in the cultural context of the breakfast meal itself. The timing and location of breakfast can also vary widely across countries and population subgroups.

This international project will be based on multiple dietary intake databases that range from 1-day 24 h recall to 7-day diet records that are publicly available. These dietary surveys vary in data collection procedures and are linked to different national nutrient composition databases. In general, nutrient composition of foods is expressed in g, mg, or mcg per 100 g edible portion. However, not all nutrients are available for all countries; the inclusion of added, free and total sugars can be problematic. Multiple standards for nutrient adequacy exist, ranging from Codex values, to values set by the European Union, the World Health Organization and local health regulatory agencies, including the Food and Drug Administration in the US. Whereas nutrient values in the US are typically expressed per serving size, nutrient labels in the EU are generally based on 100 g reference amounts.

Notwithstanding these variations, the project will seek to harmonize the analysis of national databases to the maximum possible and to present the data on nutrient intake in such a manner to allow for cross country comparisons. A modeling component of the project will discuss how prevailing food choices for breakfast can be optimized to achieve nutrient rich breakfasts that best conform to the current dietary guidelines.

Nutrient density of the total diet will be assessed using the Nutrient Rich Foods (NRF) [26], initially developed to score nutrient density of individual foods. The present variant will be used to assess the quality of the total diet by measuring nutrient intakes per reference amount (2000 kcal). The NRF9.3 score is based on 9 qualifying nutrients and 3 disqualifying nutrients. The qualifying nutrients are protein, fiber, vitamin A, vitamin C, vitamin D, calcium, iron, potassium and magnesium. The disqualifying nutrients are saturated fat, added sugar and sodium. The NRF9.3 algorithm is the sum of the percent daily values for the 9 qualifying nutrients minus the sum of daily values of 3 disqualifying nutrients. The NRF family of nutrient density scores has been fully described in previously published research.

The overall daily intake of each nutrient for each subject will be expressed as a percentage of the reference daily intake where values for nutritional labelling will be used from EU, US and Canadian standards. Values above 100% will capped at the 100th mark. To allow for comparisons across countries, population samples will be stratified by NRF tertiles. Finally, for the upper tertile, which is indicative of the highest level of overall daily nutritional quality, the nutrient intakes at breakfast will be determined. These values will be used to guide quantitative dietary guidelines for breakfast.
